# Screening for Major Depressive Disorder with the Patient Health Questionnaire (PHQ-9 and PHQ-2) in an Outpatient Clinic Staffed by Primary Care Physicians in Japan: A Case Control Study

**DOI:** 10.1371/journal.pone.0119147

**Published:** 2015-03-19

**Authors:** Keiko Suzuki, Shima Kumei, Masumi Ohhira, Tsukasa Nozu, Toshikatsu Okumura

**Affiliations:** 1 Department of General Medicine, Asahikawa University Hospital, Asahikawa, Hokkaido, Japan; 2 Department of General Internal Medicine, Asahikawa City Hospital, Asahikawa, Hokkaido, Japan; 3 Department of Regional Medicine and Education, Asahikawa University Hospital, Asahikawa, Hokkaido, Japan; University of Iowa Hospitals & Clinics, UNITED STATES

## Abstract

**Objective:**

The Patient Health Questionnaire (PHQ-9) is a self-report questionnaire commonly used to screen for depression, with ≥8–11 generally recommended as the cut-off. In Japan, studies of the validity of the PHQ-9 and PHQ-2 have been limited. In this study, we examined the utility of the PHQ-9 and PHQ-2 at an outpatient clinic in a Medical University Hospital in Japan.

**Methods:**

New consecutive outpatients were included in the study. We administered the PHQ-9 to 574 patients, and acquired complete PHQ-9 and PHQ-2 data for 521 patients. Major depressive disorders were diagnosed according to the DSM-IV-TR.

**Results:**

Forty-two patients were diagnosed with major depressive disorders. The mean PHQ-9 (15.7) and PHQ-2 (3.8) scores of the patients with major depressive disorders were significantly higher than the scores of the patients without depression (6.0 (PHQ-9) and 1.8 (PHQ-2)). The best cut-off points for the PHQ-9 and PHQ-2 summary scores were ≥11 (sensitivity 0.76, specificity 0.81) and ≥3 (sensitivity 0.76, specificity 0.82), respectively. No relationship was observed between the age and PHQ-9 scores.

**Conclusion:**

The PHQ-9 and PHQ-2 were useful instruments for screening for major depressive disorders. The best cut-off point for the PHQ-9 summary score should be ≥11 to detect depression in the primary care setting in Japan.

## Introduction

Depression is a mental illness that is associated with disability and a reduced quality of life for the person with the disorder [1]. Patients with unrecognized depression consult with their physician more frequently than those without, and consume greater health care resources [1]. The World Health Organization (WHO) Psychological Problems in General Health Care study reported that primary care physicians diagnosed major depression in only 42% of adult patients who had the condition [2]. Two-thirds of primary care patients with depression presented with somatic symptoms (eg, headache, back problems or chronic pain), making the detection of depression more difficult [3]. Improvements in detection can lead to earlier treatment, and treatment of major depressive disorders is thought to result in improved outcomes, such as a better quality of life, better work life and minimization of the risk of suicide [4]. These findings suggested that an easy and reliable method to detect depression should be used routinely, especially in the primary care setting.

The Patient Health Questionnaire (PHQ-9) is a self-report questionnaire consisting of nine questions asking about depression symptoms, and is commonly used to screen for depression, with a score of 8–11 recommended as the cut-off score, but the optimal cut-off score may differ depending on the setting [5]. The PHQ-2 is comprised of the first two questions from the PHQ-9 For example, sensitivity and specificity of the PHQ-2 for diagnosing major depression were 86% and 76% (cut-off point ≥2) and 61% and 92% with (cut-off point ≥3); for the PHQ-9, they were 74% and 91% (cut-off point ≥10) in New Zealand [6]. In the Unites States, sensitivity and specificity of the PHQ-2 for diagnosing major depression were 91% and 65% (cut-off point ≥1) and 61% and 92% with (cut-off point ≥3); for the PHQ-9, they were 54% and 90% (cut-off point ≥10) [7]. On the other hand, studies of the validity of the PHQ-9 and PHQ-2 have been limited in Japan. Inagaki et al. reported that a PHQ-9 cut-off score of ≥5 (sensitivity 0.77, specificity 0.95) and a PHQ-2 cut-off score of ≥3 was useful for detected depression in the primary care center of a rural Japanese hospital [8]. Inoue et al. reported that the optimal cut-off score was ≥14 (sensitivity 0.86, specificity 0.67) for “current major depressive episodes” in a clinic specializing in psychiatric care [9]. Therefore, a clear cut-off score for the PHQ-9 has not yet been established in Japan. In this study, we examined the utility of the PHQ-9 and PHQ-2 for detecting depression at an outpatient clinic in a Medical University Hospital in Japan.

## Materials and Methods

### Study design and participants

From October 2013 to July 2014, consecutive outpatients who visited the Department of General Medicine, Asahikawa Medical University Hospital, as new patients were included in the study. Asahikawa Medical University Hospital is located in Asahikawa City, which has a population of approximately 350,000 in the middle of Hokkaido Island, in the northernmost part of Japan. The hospital has 602 beds, and approximately 250 doctors work at the hospital to cover almost all medical problems. Among them, there are five primary care physicians working in the Department of General Medicine.

For ethical reasons, we excluded patients who couldn’t answer the PHQ-9 questionnaire since they were critically physically ill and needed emergency treatments. The PHQ-9 is a self-report questionnaire consisting of nine questions asking about symptoms of depression. The Japanese version of the PHQ-9 [10] was administered to patients who agreed to participate in this study and provided written informed consent before consultation.

We diagnosed major depressive episodes using the Japanese version of the Major Depression Episode module of the Mini-International Neuropsychiatric Interview (MINI)[11]. The MINI is a short, structured diagnostic interview used as a tool to diagnose DSM-IV disorders, and the MINI Japanese version had reliably and validly for making DSM-III-R diagnoses, and can be performed in less than half of the time required for the Structured Clinical Interview for the DSM- III-R [11] Major depressive disorders were diagnosed according to the DSM-IV-TR. This study was approved by the ethics committee of Asahikawa Medical University Hospital in Japan.

### Data analysis

To investigate the cut-off scores of the PHQ-2 and PHQ-9 for major depressive disorders, we generated receiver operating characteristic (ROC) curves, and calculated the area under the curves (AUC). The ROC curve plots the true positive rate versus the false positive rate over a range of cut-off values. It is considered that the best cut-off point is at or near the “shoulder” of the ROC curve, because as the sensitivity is progressively increased, there is little or no loss in specificity until very high levels of sensitivity are achieved. We calculated the sensitivity, specificity, positive predictive value (PPV), negative predictive value (NPV), odds ratio, positive and negative likelihood ratios and overall accuracy of the PHQ-9 and PHQ-2. In order to test for an association between age and the PHQ-9 summary scores of the depressed and non-depressed subjects, we calculated Pearson’s product-moment correlation. Student’s *t*-test was used to compare whether there were significant differences between the depressed and non-depressed subjects in the age, PHQ-9 total scores and PHQ-2 scores.

## Results

A total of 650 outpatients who visited the Department of General Medicine, Asahikawa Medical University Hospital as new patients were included in the study. For ethical reasons, we excluded 76 patients who were critically ill from filling out the PHQ-9 forms, or who didn’t agree to provide consent for participation. We administered the PHQ-9 to 574 patients and acquired complete PHQ-9 data for 521 patients. The age of the 521 patients was 51.0 ± 19.4 (mean ± SD) years old. As shown in Table 1, the ICD-10 diagnoses of these 521 patients were widely distributed in almost all disease fields. Among them, 42 patients (8.1%) were diagnosed to have a major depressive disorder. The PHQ-9 and PHQ-2 scores in patients with depression were significantly higher than those in patients without depression (Table 2), strongly supporting that the PHQ-9 and PHQ-2 scores are capable of discriminating depression from other conditions.

**Table 1:** International Classification of Disease (ICD)-10 Diagnoses in 521 patients.

Chapter	Title	n	n/Total
I	Certain infectious and parasitic diseasese	89	0.17
II	Neoplasms	3	0.01
III	Disease of the blood and blood-forming organs and certain disorders involving the immune mechanism	6	0.01
IV	Endocrine, nutritional and metabolic diseases	17	0.03
V	Mental and behavioural disorders	128	0.25
VI	Disease of the nervous system	55	0.11
VII	Disease of the eye and adnexa	0	0.00
VIII	Disease of the ear and mastoid process	9	0.02
IX	Disease of the circulatory system	23	0.04
X	Disease of the respiratory system	15	0.03
XI	Disease of the digestive system	32	0.06
XII	Disease of the skin and subcutaneous tissue	22	0.04
XIII	Disease of the musculoskeletal system and connective tissue	78	0.15
XIV	Disease of the genitourinary system	20	0.04
XV	Pregnancy, childbirth and the puerperium	0	0.00
XVI	Certain conditions originating in the perinatal period	0	0.00
XVII	Congenital malformations, deformations and chromosomal abnormalities	0	0.00
XVIII	Symptons, sings and abnormal clinical and laboratory findings, not elesewhere classified	1	0.00
XIX	Injury, poisoning and certain other consequences of external causes	12	0.02
XX	External causese of morbidity and mortality	0	0.00
XXI	Factors influencing health status and contact with health services	0	0.00
XXII	Codes for special purposes	0	0.00
	Others	11	0.02
	Total	521	1

**Table 2:** PHQ-9 and PHQ-2 scores in patients with major depression.

	non depression	depression	
N	479	42	
Age	51.2±19.5	48.4±18.3	
PHQ-9	6.0±5.2	15.7±6.2	*
PHQ-2	1.3±1.5	3.8±1.8	*

* p < 0.01 mean ± SD

**Fig 1:**
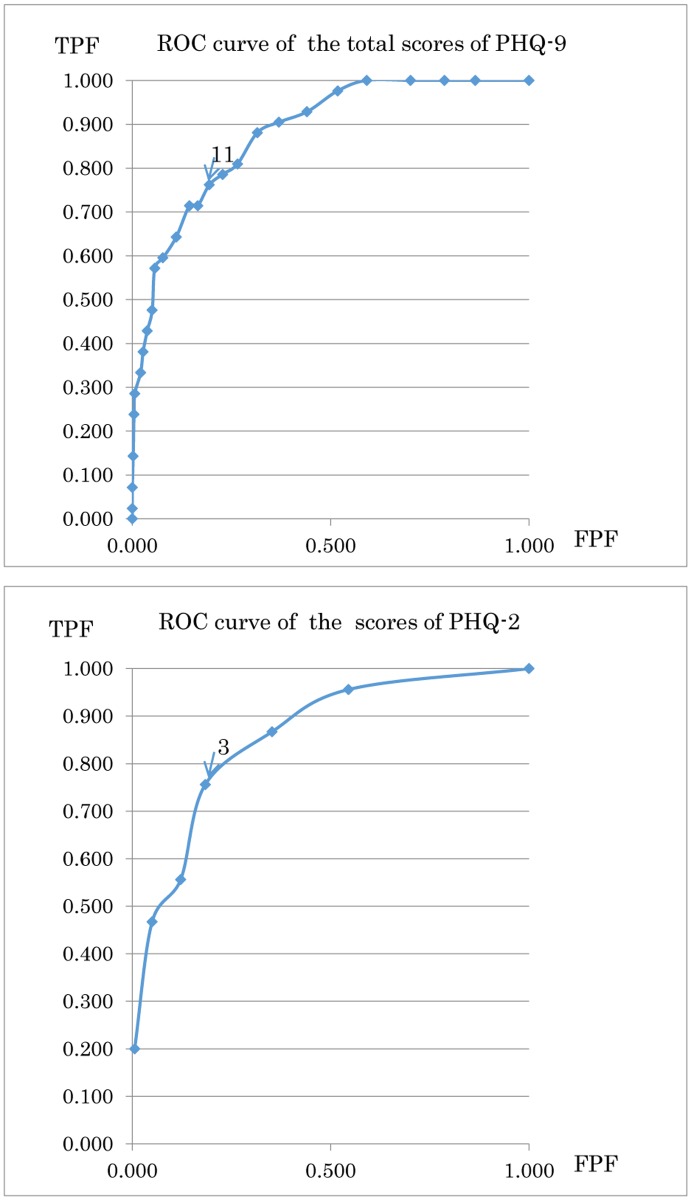
The ROC curve of the total scores of the PHQ-9 and PHQ-2. TPF: True-positive fraction, FPF: False-positive fraction

Table 3 shows the sensitivity and specificity at different cut-off scores for the PHQ-9 and PHQ-2 summary scores in the patients with a major depressive disorder. Fig. 1 shows the ROC curves of the cut-off points of the PHQ-9 and PHQ-2 summary scores for major depressive disorders based on the sensitivity and specificity of the PHQ scores. The areas under the ROC curves of PHQ-9 and PHQ-2 were 0.880 and 0.845, respectively. The best cut-off points for the PHQ-9 and PHQ-2 summary scores determined to the nearest point from the “shoulder” of the ROC curve were 11 (sensitivity 0.762, specificity 0.806) and ≥3 (sensitivity 0.762, specificity 0.814), respectively. The overall accuracy, sensitivity, specificity, positive predictive value, negative predictive value, odds ratio, positive likelihood ratio and negative likelihood ratio when the cut-off point was set at > 11 (PHQ-9) and > 3 (PHQ-2) are shown in Table 4, thus indicating a good screening performance.

**Table 3:** Sensitivity and specificity of PHQ-9 and PHQ-2.

Cut-off points	Sensitivity	Specificity
PHQ-9≧6	0.93	0.56
≧7	0.90	0.63
≧8	0.88	0.69
≧9	0.81	0.74
≧10	0.79	0.77
≧11	0.76	0.81
≧12	0.71	0.84
≧13	0.71	0.86
≧14	0.64	0.89
≧15	0.60	0.92
PHQ-2≧1	0.95	0.45
≧2	0.86	0.65
≧3	0.76	0.81
≧4	0.55	0.88
≧5	0.45	0.95
≧6	0.19	0.99

**Table 4:** Screening performance of the PHQ-9 and PHQ-2.

	PHQ-9	PHQ-2
Overall accuracy (95% CI)	0.81(0.77–0.84)	0.81(0.78–0.85)
Sensitivity (95% CI)	0.76(0.61–0.88)	0.76(0.61–0.88)
Specifisity (95%CI)	0.81(0.77–0.85)	0.82(0.78–0.85)
Positive Predictive Value (95% CI)	0.26(0.19–0.35)	0.27(0.19–0.36)
Negative Predictive Value (95% CI)	0.97(0.95–0.99)	0.98(0.96–0.99)
Odds ratio (95% CI)	13.83(6.56–29.17)	14.22(6.74–30.00)
Positive likelihood ratio	4.06	4.15
Negative likelihood ratio	0.29	0.29
True positive, n	32	32
False positive, n	90	88
False negative, n	10	10
True negative, n	389	391

**Fig 2:**
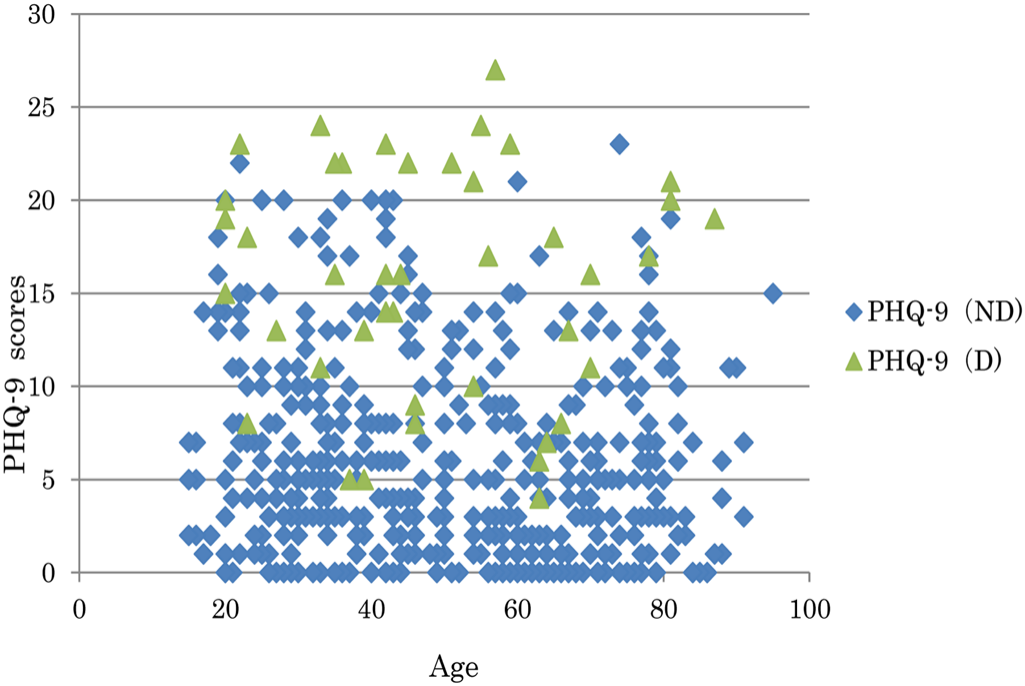
The relationship between the age and PHQ-9 total scores of the patients. ND: non-depressed, D: depressed.

Fig. 2 shows the relationship between the age and the PHQ-9 total scores. The Pearson’s product-moment correlation revealed no correlation between age and the PHQ-9 total scores in not only depressed patients, but also in patients without depression.

## Discussion

Some meta-analyses of the PHQ-9 scores for diagnosing depression have been reported [5, 12]. For example, Laura et al. reported that the PHQ-9 had acceptable diagnostic properties for detecting major depressive disorder for cut-off scores between ≥8 and 11 [5]. Gilbody et al. reported that the PHQ-9 (cut-off point ≥10) is an acceptable instrument for detecting major depressive disorders in primary care patients (sensitivity 0.80, specificity 0.92) [12].

In Japan, some previous studies have described the cut-off points of the PHQ-9. Inagaki et al. reported that the best PHQ-9 cut-off point was ≥5 for depression in the primary care setting at a Japanese rural hospital (mean age of patients: 73.5 years old)[8]. In the present study, the PHQ-9 score of patients without depression was 6.0 ± 5.2 (mean ± SD), suggesting that over a half of the patients without depression would be included in patients considered to have depression if the cut-off point of the PHQ-9 was set at ≥5. We would therefore suggest that the best PHQ-9 cut-off point (≥5) proposed by Inagaki et al. would be too small. As demonstrated in the present report, we calculated that the best cut-off points for the PHQ-9 total score was ≥11 (sensitivity 0.76, specificity 0.81) in an outpatient clinic at a Japanese Medical University Hospital (mean age 51.7). This cut-off point was similar to that of the meta-analysis of studies from other countries, suggesting that the best cut-off point for the PHQ-9 total score should also be around 10 in Japan.

It was considered that the different cut-off points in Japanese studies may have been due to the differences in the mean age of the study populations. With regard to this point, we demonstrated in the present study that there was no relationship between age and the PHQ-9 total scores in not only depressed patients, but also the patients without depression. These results may suggest that the age difference is not a key factor to explain the different finding with regard to the best cut-off point for the PHQ-9 score between the present study and the study by Inagaki et al. [8]. Further studies are needed to explain the differences and to confirm the best cut-off point.

In our study, the PHQ-2 (cut-off point ≥3) had a sensitivity of 0.76 and specificity of 0.76, which were similar to those of the PHQ-9 (sensitivity 0.81, specificity 0.76, cut-off point ≥11). Lowe et al. reported that the PHQ-2 (cut-off point ≥3) had a sensitivity of 0.87 and a specificity of 0.78 for major depressive disorder in several outpatient clinics [13]. In Japan, Inagaki et al. reported that the PHQ-2 (cut-off point ≥3) had a sensitivity of 0.77 and a specificity of 0.95, and mentioned that the PHQ-2 may be preferred in screening for patients with major depression in internal medicine outpatient clinics [8]. These findings clearly indicate that the PHQ-2 cut-off point should also be ≥3 in Japan.

## Conclusion

The PHQ-9 and PHQ-2 are both useful instruments for screening for major depressive disorder in an outpatient clinic in a Japanese hospital. In this study, the PHQ-2 (cut-off point ≥3) and the PHQ-9 (cut-off point ≥11) should be applied to identify patients with depression in the primary care setting in Japan.
